# *Babesia divergens*–like Infection, Washington State

**DOI:** 10.3201/eid1004.030377

**Published:** 2004-04

**Authors:** Barbara L. Herwaldt, Guy de Bruyn, Norman J. Pieniazek, Mary Homer, Kathryn H. Lofy, Susan B. Slemenda, Thomas R. Fritsche, David H. Persing, Ajit P. Limaye

**Affiliations:** * Centers for Disease Control and Prevention, Atlanta, Georgia, USA; † University of Washington, Seattle, Washington, USA; ‡ Corixa Corporation, Seattle, Washington, USA; § Washington State Department of Health, Olympia, Washington, USA

**Keywords:** babesiosis, *Babesia divergens*, *Babesia microti*, *Babesia odocoilei*, EU1, MO1, WA1, CA1, 18S rRNA gene, Washington State, clindamycin, quinine

## Abstract

Most reported U.S. zoonotic cases of babesiosis have occurred in the Northeast and been caused by *Babesia microti*. In Washington State, three cases of babesiosis have been reported previously, which were caused by WA1 (for “Washington 1”)-type parasites. We investigated a case of babesiosis in Washington in an 82–year-old man whose spleen had been removed and whose parasitemia level was 41.4%. The complete 18S ribosomal RNA gene of the parasite was amplified from specimens of his whole blood by polymerase chain reaction. Phylogenetic analysis showed the parasite is most closely related, but not identical, to *B. divergens* (similarity score, 99.5%), a bovine parasite in Europe. By indirect fluorescent-antibody testing, his serum reacted to *B. divergens* but not to *B. microti* or WA1 antigens. This case demonstrates that babesiosis can be caused by novel parasites detectable by manual examination of blood smears but not by serologic or molecular testing for *B. microti* or WA1-type parasites.

Hundreds of zoonotic cases of babesiosis have been reported in the United States, approximately 30 in Europe, and a few elsewhere (*1*–*14*). Most of the reported U.S. cases have been caused by *Babesia microti*, a parasite of small mammals transmitted by *Ixodes scapularis* ticks, and have occurred in the Northeast or, less commonly, the upper Midwest (*4*).

Few zoonotic cases of babesiosis have been reported in the western United States (*7*–*11*). Specifically, in Washington State, only three cases, two presumably tick-borne and one associated with blood transfusion, have been reported previously (*7*,*8*,*10*). The index tick-borne case occurred in 1991 in Klickitat County, in south-central Washington (*7*,*8*). The other two cases occurred in 1994: one in a person transfused with infected erythrocytes and the other in the implicated blood donor, who lived in King County, in the western foothills of the Cascade Mountains (*10*). All three of these cases were caused by WA1 (for “Washington 1”)-type parasites (*1*,*2*,*7*–*10*,*14*) (Figure 1)*.* However, the etiologic agent of the case of babesiosis in Washington in 2002 that we describe here is most closely related, by molecular criteria, to *B. divergens*, a bovine parasite in Europe (*5*,*6*).

**Figure 1:**
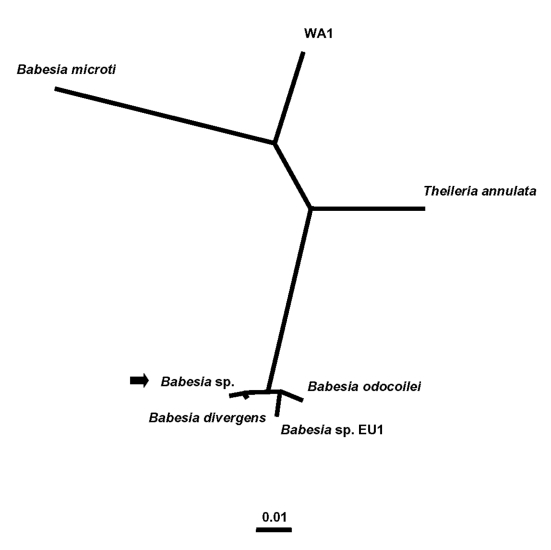
Unrooted phylogenetic tree for the complete 18S rRNA gene of selected *Babesia* spp. The tree was computed by using the quartet puzzling maximum likelihood method of the TREE-PUZZLE program. The scale bar indicates an evolutionary distance of 0.01 nucleotide substitutions per position in the sequence. The GenBank accession numbers for the sequences used in the analysis are as follows: *B*. *divergens* (6), AY046576; *B*. *odocoilei*, AY046577; *Babesia* sp. EU1 (6), AY046575; the *Babesia* sp. from the patient in Washington State, AY274114 (see arrow); *B. microti*, U09833; WA1, from the index case of infection with WA1-type parasites (7,8), AF158700; and *Theileria annulata*, M64243.

## Methods

### Serologic Testing

Serum specimens were tested at the Centers for Disease Control and Prevention (CDC), in serial fourfold dilutions, by indirect fluorescent-antibody (IFA) testing, for reactivity to *B. microti* (*15*), WA1 (*7*), and *B. divergens* antigens (*6*). The antigen sources were human isolates of *B. microti* and WA1 and a bovine isolate of *B. divergens* (Purnell strain [*16*]), which had been passaged in gerbils (Mongolian jirds; *Meriones unguiculatus*) and adapted to culture in bovine erythrocytes (*17*).

### Animal Inoculation

To attempt to obtain an isolate of the parasite that infected the patient (hereafter referred to as the patient’s parasite), whole blood specimens, collected from him in tubes containing the anticoagulant disodium EDTA, were injected into hamsters (*Mesocricetus auratus*) and jirds. Animal experimentation guidelines were followed. Both hamsters and jirds are competent hosts for *B. microti* and WA1-type parasites, and jirds are competent hosts for *B. divergens* (*18*). No pretreatment specimens were available. Refrigerated specimens collected on July 31, 2002 (the date antibabesial therapy was begun [Table 1]), and August 1 were each injected intraperitoneally into two hamsters (0.25 mL and 0.5 mL/animal, respectively) on August 2. A specimen obtained on September 10 (15 days after therapy was discontinued) was injected intraperitoneally into two jirds (1 mL/animal) the next day. The animals were monitored for parasitemia, weekly for 8 weeks, by examination of Giemsa-stained smears of blood obtained by tail snip (hamsters) or toenail clip (jirds). Blood obtained from the animals after the 8-week monitoring period was examined by polymerase chain reaction (PCR; see next section).

**Table 1:** Clinical data on selected dates for a patient in Washington State infected with a *Babesia divergens*-like parasite, 2002^a^

Date	Temperature (°C)	Hematocrit (%)^b^	Leukocyte count (10^9^/L)	Platelet count (10^9^/L)	Parasitemia level (%)^c^	Creatinine level (mg/dL)^d^	Total/direct bilirubin levels (mg/dL)	Lactate dehydrogenase level (U/L)	Comments	
July 30	38.5	43	19.6	34	25.5 ^c^	8.5	10.2	4,283	Admitted to a community hospital	
July 31^e^		40	21.2^f^	21	41.4 ^c^	10.3	8.2/2.9		Babesiosis diagnosed; antibabesial therapy started (see text)^g^	
July 31^h^	39.9	36	18.5	25	28.2	11.1^i^	9.9/3.1	6,674	6 U platelets transfused	
August 1	37.7	27	21.6	57	24.7	6.4	6.7/2.3	2,898	Began hemodialysis; 2 U packed erythrocytes transfused	
August 2	36.9	32	22.1	67	17.9	4.2	3.3/2.1	5,802		
August 3	37.5	29	17.2	96	13.6	7.3		2,423		
August 4	36.9	27	10.4	110		6.4	1.3/0.7			
August 5	37.6	26	9.9	135	11.9	9.0				
August 6	37.5	24	9.5	149	13.2	11.0			2 U packed erythrocytes transfused	
August 7	37.5	30	9.9	149		8.8				
August 8	37.9	30	9.2	204	7.7	11.2				
August 9	36.7	28	9.2	161^j^		8.2			Discharged home	
									
September 13		28	7.3	179		2.8				
November 1		39	6.0			1.7				

^a^Normal ranges for laboratory values at community hospital (July 30–31): creatinine, 0.7–1.5 mg/dL; total bilirubin, 0.1–1.0 mg/dL; lactate dehydrogenase, 100–200 U/L. Normal ranges at University of Washington Medical Center (July 31–Aug. 9): creatinine, 0.3–1.2 mg/dL; total bilirubin, 0.1–1.0 mg/dL; direct bilirubin, 0.0–0.3 mg/dL; lactate dehydrogenase, 0–190 U/L. ^b^Hematocrit normalized to values of 42% (Feb. 3, 2003) and 45% (June 27, 2003). ^c^All parasitemia levels were determined by the same person at the Centers for Disease Control and Prevention. Level for July 30 was determined from a peripheral smear made the next day (i.e., blood not fresh). The other pretreatment parasitemia level (i.e., from July 31 at community hospital) was determined from a smear made from fresh blood. ^d^Baseline and posthospitalization creatinine values include: 1.6 mg/dL (Sept. 2001), 1.7 mg/dL (Feb. 3, 2003), and 1.6 mg/dL (June 27, 2003). ^e^Data obtained at community hospital. ^f^Leukocyte differential: 74% segmented neutrophils, 9% lymphocytes, 8% atypical lymphocytes (including immunoblasts and plasmacytoid forms), 7% monocytes, 1% eosinophils, and 1% metamyelocytes. ^g^On Aug. 1, dosage regimen changed from clindamycin (1.2 g twice daily, by IV infusion) and quinine sulfate (650 mg thrice daily, by mouth), to clindamycin (600 mg four times daily, by IV infusion) and quinine (650 mg daily, by mouth). After patient was discharged from hospital on Aug. 9, therapy continued through Aug. 26; regimen: clindamycin (600 mg four times daily, by mouth) and quinine (650 mg daily, by mouth). Patient also treated with erythropoietin until Nov. 2002. ^h^Data obtained at University of Washington Medical Center. ^i^Dipstick analysis of urine specimen showed 3+ protein, 3+ blood, 3+ bilirubin, and 1+ ketones; and was positive for nitrites and leukocyte esterase. Microscopic examination showed granular casts and 1+ leukocytes. Culture demonstrated >10^5^ colonies of *Escherichia coli* per milliliter of urine. The urinary tract infection was treated with ceftriaxone. ^j^Value could be inaccurate; platelets clumped on blood smear.

### DNA Extraction, Amplification, and Sequencing

DNA was extracted at CDC from whole blood specimens collected from the patient in EDTA tubes, by using the QIAamp DNA Blood Mini Kit (Qiagen Inc., Valencia, CA); the DNA was stored at 4°C. The complete 18S ribosomal RNA (18S rRNA) gene from the patient’s parasite was amplified by PCR, with a pair of apicomplexan 18S rRNA-specific primers: CRYPTOF (5′-AACCTGGTTGATCCTGCCAGT-3′) and CRYPTOR (5′- GCTTGATCCTTCTGCAGGTTCACCTAC-3′). PCR was conducted with AmpliTaq Gold DNA Polymerase (Applied Biosystems, Foster City, CA). The conditions for PCR included 95ºC for 15 min, followed by 45 cycles of denaturation at 94°C for 30 s, annealing at 65°C for 30 s, and extension at 72°C for 1.5 min. Final extension was done at 72°C for 9 min, followed by a hold step at 4°C. The amplification product was purified by using the StrataPrep DNA Purification Kit (Stratagene, La Jolla, CA).

Both strands of the PCR product were sequenced by using a set of internal primers. Sequencing reactions were performed with the ABI PRISM BigDye Terminator Cycle Sequencing Kit (Applied Biosystems), and reactions were analyzed on the ABI 3100 automatic DNA sequencer (Applied Biosystems). The resulting sequences were assembled by using the program SeqMan II (DNASTAR, Inc., Madison, WI). The GenBank accession number for the sequence obtained by these methods for the patient’s parasite is AY274114.

### Phylogenetic Analysis

The complete sequences of the 18S rRNA genes for *B*. *divergens*; *B*. *odocoilei*, a parasite of white-tailed deer (*Odocoileus virginianus*) (*17*,*19*); *Babesia* sp. EU1 (for “European Union 1”) (*6*); *B. microti*; the WA1 parasite from the index case of such infection (*7*,*8*); and *Theileria annulata* were retrieved from the GenBank database (Figure 1). The sequences were aligned with the sequence for the patient’s parasite by using the program Clustal W, version 1.83 (*20*). Phylogenetic analysis was performed with the following programs: the PHYLIP package, which includes versions 3.573c of CONSENSE, DNADIST, DNAML, NEIGHBOR, and SEQBOOT (*21*); and version 5.1 of TREE-PUZZLE (*22*). The phylogenetic trees inferred by these programs were drawn with the program TreeView, version 1.6.6 (*23*).

## Case Report

On July 30, 2002, an 82-year-old man in Kitsap County (a peninsula of estuarine lowlands in Puget Sound, in western Washington), was admitted to a community hospital. During the previous 4 days, he had noted the gradual onset of chills (without fever), weakness, malaise, anorexia, dysphagia, marked thirst, and urinary dribbling. Until April or May 2002, he had been an avid jogger. Although he had not had the energy to jog thereafter, he had continued to walk his dog daily; they walked on a road around a lake that abutted his backyard, which he kept “natural,” and on a neighbor’s wooded property. He had an outdoor cat; had occasionally noted deer and (in previous years) voles or shrews in his yard; did not live near cattle; and had not traveled outside of Kitsap County or Mason County, the adjacent southern county, in the previous couple of years. He had not noted ticks on his body or received blood transfusions. His medical history included longstanding hypertension; secondary renal insufficiency; and incidental splenectomy in approximately 1990, when his distal pancreas, which had a benign mass, was removed.

When he was hospitalized on July 30, his temperature was 38.5°C; pulse, 76 beats/min; blood pressure, 168/94 mm Hg; and respiratory rate, 18 breaths/min. He had scleral icterus and dry mucous membranes from severe dehydration. The salient laboratory data and information about his clinical course are provided in Table 1. Initial laboratory testing showed many abnormalities, including marked thrombocytopenia and elevated values of creatinine, bilirubin, and lactate dehydrogenase.

**Figure 2:**
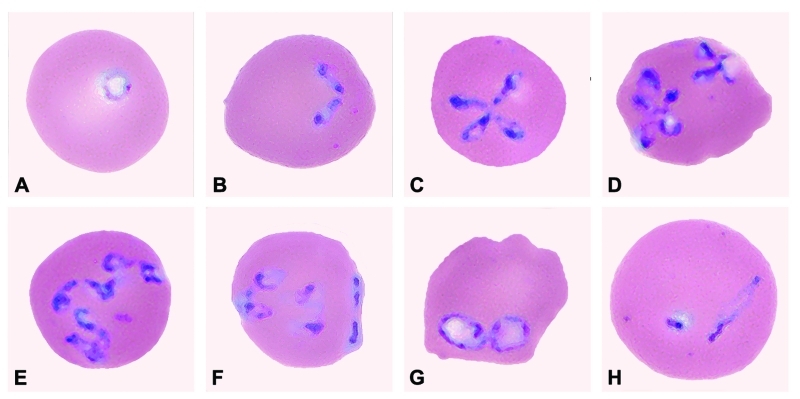
Panel of *Babesia*-infected erythrocytes photographed from pretreatment, Wrights-Giemsa–stained smears of fresh blood obtained from the patient on July 31, 2002. The mean corpuscular volume of the erythrocytes was 103 (normal range 80–100 μm^3^). Note the multiply infected erythrocytes; the pleomorphism of the parasite; and the obtuse (divergent) angle formed by some of the paired structures, which, like the form in (F), is characteristic of *B. divergens* and related parasites isolated from various wild ruminants. The forms of the parasite shown in the panel include: (A) ring-like trophozoite; (B) paired merozoites; (C) maltese-cross (tetrad); (D) various dividing forms; (E) multiple merozoites; (F) appliqué (accolé) form on right border of the erythrocyte; (G) and (H) degenerate (crisis) forms. A glass slide of a peripheral blood smear from July 31 has been deposited in the U.S. National Parasite Collection, Beltsville, Maryland; the accession number (USNPC #) for the slide is 093041.00.

Babesiosis was diagnosed, when a peripheral smear made on July 31 from blood collected on July 30 was noted to have intraerythrocytic, ring-like trophozoites; the level of parasitemia on a smear of fresh blood from July 31 was 41.4% (Table 1; Figure 2). On July 31, antibabesial therapy was begun at the community hospital; he received one dose of clindamycin (1,200 mg, by intravenous infusion) and one dose of quinine sulfate (975 mg, by mouth; the intended—i.e., ordered—dose was 650 mg).

Later on July 31, he was transferred to the University of Washington Medical Center. He continued therapy with clindamycin and quinine, with periodic adjustments of the dosage regimen (Table 1 footnote). In addition, he was transfused with 6 U of platelets and 4 U of packed erythrocytes. His acute renal failure (Table 1) was treated with hemodialysis; it was attributed to acute tubular necrosis from dehydration and intravascular hemolysis, superimposed on chronic renal insufficiency from hypertensive nephrosclerosis. His hospital course was also notable for laboratory evidence, on August 1, of asymptomatic, subendocardial ischemia and for pulmonary edema.

After he was discharged from the hospital on August 9, he continued antibabesial therapy through August 26 (Table 1) and hemodialysis through early September. He resumed jogging and walking in early October 2002 and had remained well as of July 2003.

## Results

### Serologic Testing and Animal Inoculation

The patient’s serum did not react to *B. microti* or WA1 antigens but showed marked IFA reactivity to *B. divergens* antigens, which slowly decreased during the 9-month monitoring period (Table 2). Attempts to obtain an isolate of the patient’s parasite, by injecting specimens of his blood into hamsters and jirds, were unsuccessful (Table 2 footnote). PCR analysis of blood from the inoculated animals, after they had been monitored for 8 weeks, was negative.

**Table 2:** Results of testing of serial specimens obtained from a patient in Washington State infected with a *Babesia divergens*-like parasite^a^

Date^b^	Examination of smears of whole blood for *Babesia* parasites	IFA titers to *B. divergens* antigens^c^	PCR
July 31, 2002	Positive (parasitemia level, 28.2%)	1:64	Positive
September 10	NPF	1:1,024	Positive
October 25	NPF	1:1,024	Positive
November 8	NPF	1:256	Negative
December 12	NPF	1:256	Negative
April 14, 2003	NPF	1:256	Negative

^a^IFA, indirect fluorescent antibody; PCR, polymerase chain reaction; NPF, no parasites found. ^b^See Table 1 for values of other laboratory testing performed on or near dates listed here. Patient received antibabesial therapy from July 31 (before July 31 specimen obtained) through August 26, 2002. Hamsters injected with patient’s blood obtained July 31 and August 1 did not become demonstrably parasitemic, nor did jirds injected with blood from September 10. ^c^All of the serum specimens were tested at the Centers for Disease Control and Prevention on same day.

### Molecular Findings

Amplification of the complete 18S rRNA gene of the patient’s parasite yielded a specific product of approximately 1,700 bp. Sequence analysis showed that the gene was 1,728 bases long. The sequences of the PCR products from three blood specimens from the patient (Table 2), each processed separately, were identical. The DNA sequence also was verified by staff in an independent laboratory, who had never worked with *B. divergens* or DNA extracted from it.

BLAST search confirmed that the sequence for the patient’s parasite was not identical to any complete 18S rRNA sequence in GenBank. The highest similarity score (99.5%) was with *B*. *divergens* (GenBank no. AY046576); the sequences for *B. divergens* and the patient’s parasite differ by eight bases.

In phylogenetic analysis (Figure 1), the patient’s parasite clusters together with *B*. *divergens*. This group forms a sister group to a cluster that includes *B*. *odocoilei* and *Babesia* sp. EU1 (*6*). The clustering of the organisms was the same, regardless of the taxonomic method used. The alignment of the sequences used to construct the phylogenetic tree (Figure 1), after columns with gaps and unresolved characters were removed, had 1,651 columns; the bases in 255 columns differed among the *Babesia* spp. included in the analysis. The alignment may be requested from the authors. Serial PCR and IFA data (Table 2) showed that the patient had subpatent parasitemia and a persistently high IFA titer (1:1,024) for at least 2 months after his antibabesial therapy was stopped.

## Discussion

This case of babesiosis had several unusual features. First, it occurred in Washington State, rather than in the Northeast, where most of the reported U.S. cases of zoonotic babesiosis have occurred. Our case raises the count for reported cases of babesiosis in Washington from three to four (one bloodborne and three presumably tick-borne cases). Second, the case was caused by a parasite most closely related, by molecular criteria, to *B. divergens*, a European bovine parasite (*24*), rather than to WA1-type parasites, which caused the three previously reported cases of babesiosis in the state. Third, the patient whose case we describe survived, despite having multiple risk factors for severe babesiosis and death: he was elderly (82 years old), was asplenic, had a high level of parasitemia (41.4%), and had multiorgan dysfunction that included renal failure.

Few cases of babesiosis in the western United States have been reported previously; all occurred in Washington or California. They include two tick-borne cases in California in 1966 (*25*) and 1979 (*26*), as well as seven tick-borne and two blood-transfusion–associated cases in California and Washington from 1991 to 2000 (*7*–*11*). Whereas the etiologic agents of the cases in 1966 and 1979 were not determined, the last nine cases were caused by the CA1- and WA1-types of *Babesia*- (or *Babesia*-like) piroplasms, which are distinct from each other but in the same phylogenetic group. Although the appropriate position for this clade in phylogenetic analyses of the piroplasms remains unclear (*14*), the position is remote from *B. microti* and *B. divergens* (*11*,*14*) (Figure 1).

The molecular characterization of our patient’s parasite (Washington, 2002) showed that the sequence for the complete 18S rRNA gene differs by eight bases from that of *B. divergens* (similarity score, 99.5%). In addition, serologic (Table 2) and morphologic (Figure 2) data support the conclusion that the patient was infected with a *B. divergens*–like parasite.

*B. divergens* infects cattle in Europe but has never been reported to do so in the United States. The parasite that caused the index bovine case of *B. divergens* infection, which was described in 1911 (*27*), is unavailable for molecular characterization. However, the DNA sequences of the complete 18S rRNA gene for bovine isolates of *B. divergens* from three European countries (*6*) and an isolate from an infected *Ixodes ricinus* tick from another European country (N.J. Pieniazek, unpub data) are identical; sequence data for the complete 18S rRNA gene of *B. divergens* from its tick vector have never previously been reported.

*B. divergens* has traditionally been considered not only a bovine parasite but also the etiologic agent of most of the reported zoonotic cases of babesiosis in Europe; the cases purportedly caused by *B. divergens* typically have occurred in asplenic persons who died if not appropriately and expeditiously treated (*5*). The type and quality of the evidence used to support the conclusion that *B. divergens* caused the zoonotic cases have varied markedly (*5*,*6*). To our knowledge, sequence data for the 18S rRNA gene have been reported for only two such cases. For one of the two cases (*28*), sequence data for the complete 18S rRNA gene were reported (EMBL data base no. AJ439713; data for 1,728 bases), which were not identical to the sequence for bovine *B. divergens* (*6*). For the other case (*29*), sequence data for a portion of the gene were reported (GenBank no. AF435415; data for 369 bases). Some of the European zoonotic cases attributed to *B. divergens* infection might have been caused by EU1, the etiologic agent of the first reported zoonotic cases of babesiosis in Italy and Austria, which ocurred in 1998 and 2000, respectively (*6*). In phylogenetic analysis, EU1 is most closely related to *B. odocoilei* (*17*,*19*) and is secondarily related to *B. divergens* (31 base differences) (*6*).

Besides our case, two other U.S. zoonotic cases have been attributed to infection with *B. divergens*–like organisms, on the basis of sequence data for the 18S rRNA gene (*12*,*13*). The first, a fatal case in Missouri in 1992, occurred in a 73-year-old asplenic man, whose parasitemia level was 3%–4% (12). In the original description of the case and the etiologic agent (MO1, for “Missouri 1”) (*12*), molecular data were provided for only a 128-bp fragment (with three unresolved positions), in which MO1 and *B. divergens* have identical sequences (*6*,*12*). The other U.S. case occurred in Kentucky in 2001, in a 56-year-old asplenic man, with a parasitemia level of 30% to 35% (*13*). The sequence of the complete 18S rRNA gene of the etiologic agent reportedly differs by three bases from that for *B. divergens* (similarity score, 99.8%). However, because no GenBank accession number was provided (*13*), we do not know whether the three base differences constitute a subset of the eight we found between our patient’s parasite and *B. divergens*.

We do not know enough about the biology of the etiologic agents of these three U.S. cases attributed (by molecular criteria) to infection with *B. divergens*–like parasites, to conclude how closely related the parasites are to the European bovine *B. divergens*. Various wild ruminants in the United States and Europe have been found to be infected with parasites that are considered *B. divergens–*like in some respects (e.g., are in the same phylogenetic clade, demonstrate serologic cross-reactivity in IFA testing, have similar morphologic characteristics [Figure 2]). Some such parasites (e.g., *B. odocoilei*, a parasite of white-tailed deer [*6,17,19*]; 30 base differences from *B. divergens* [similarity score, 98.3%]) are known to be different species than *B. divergens*. However, the classification by species of some *B. divergens*–like parasites remains unresolved (*6*). For example, the sequence for the complete 18S rRNA gene for a reindeer *Babesia* sp. in Scotland differs by only four bases from that of *B. divergens* (similarity score, 99.8%). Although the organism is not known to cause overt disease in local cattle and did not infect jirds injected with several-day-old blood from infected reindeer (*30*), the biologic data available to date are not definitive. Until more parasites that are *B. divergens*–like by molecular criteria, such as the parasites that caused the three U.S. zoonotic cases, are identified and characterized in other respects, we will not know whether the parasites are synonymous with *B. divergens* or belong to a complex of related species or strains.

The public health importance of the *B. divergens*–like organism in Washington is not yet known and may take years to determine. Its biology, geographic distribution, ecology, tick vector, and animal reservoir host(s), as well as the prevalence of infection in nonhuman and human hosts, risk factors for infection and disease in humans, and clinical manifestations of infection must be further investigated. We have begun our search for the tick vector; however, no ticks were found in September 2002, after flagging for 18 person-hours near the patient’s house. Molecular analysis at CDC of DNA from 98 ticks of various species, from various animals and counties in Washington, showed that none were infected with the patient’s parasite, but 11 were infected with *B. odocoilei* (data not shown).

The clinical aspects of our patient’s case are notable, particularly the fact that he survived, despite critical illness. The extent to which host (e.g., advanced age) versus parasite factors contributed to the severity of the case are unknown. However, even *B. microti*, which traditionally has been considered less virulent than *B. divergens* in humans (i.e., *B. microti* infection often is asymptomatic or associated with mild, nonspecific symptoms), has been associated with critical illness and fatalities, particularly among elderly, asplenic, or otherwise immunocompromised, patients (*3*,*4*,*31*,*32*). His remarkably good physical condition for his age and the meticulous medical care he received likely contributed to his survival.

We cannot generalize from his case to conclude what constitutes optimal antimicrobial therapy for infection with the patient’s parasite. The one clinical trial in which the effectiveness of antimicrobial regimens for treatment of babesiosis was evaluated included only patients who were not severely ill and were infected with *B. microti* (*33*). Anecdotal data and extrapolation from the literature about treatment of malaria suggest that exchange transfusion may be beneficial for some critically ill patients, especially for those with signs of hemodynamic instability or high parasitemia levels (e.g., >10%) (*31*,*34*–*36*). The persistence of PCR positivity in our patient for at least 2 months after he completed therapy (Table 2) indicates he continued to have subpatent parasitemia, despite remaining clinically well. Persistence of PCR positivity after treatment of symptomatic cases of *B. microti* infection with clindamycin and quinine has also been reported (*37*,*38*); PCR positivity lasted a mean of 16 days in 22 such patients, about one third of whom had persistent positivity for >1 month (none for >3 months) (*37*).

The case we describe underscores several points for clinicians. First, the diagnosis of babesiosis should be considered for febrile persons with hemolytic anemia, regardless of where they live or have traveled. Second, babesiosis, which can be life threatening, can be caused by novel parasites not detected by serologic or molecular testing for *B. microti* or the WA1- or CA1-type parasites (i.e., for parasites previously recognized to cause zoonotic babesiosis in the United States). This fact underscores the importance of manual examination of smears of blood from patients who might have babesiosis. In most hospitals, blood smears are examined by machines rather than by laboratory staff, unless specific criteria are met by the patient, certain abnormalities are “flagged” by the machine, or manual examination is specifically requested. Third, thorough characterization of novel *Babesia* spp. is needed to advance our knowledge about zoonotic parasites and to facilitate development of laboratory methods for detecting such parasites in patients, participants in epidemiologic investigations, and ultimately, perhaps, blood donors. Characterization of novel *Babesia* spp. would be facilitated if clinicians with patients likely infected outside of the geographic areas known to be endemic for *B. microti* sent fresh, pretreatment, anticoagulated, whole blood specimens, by overnight mail, on wet ice packs, to a reference laboratory experienced in doing such work.
